# Role of Nuclear Motion in the Ultrafast Relaxation Dynamics of Inner-Shell Vacancies in Hydrated Pyrrole

**DOI:** 10.1021/acs.jpca.6c03998

**Published:** 2026-08-17

**Authors:** Kedong Wang, Bohui Wan, Cody L. Covington, Kálmán Varga

**Affiliations:** † School of Physics, 66519 Henan Normal University, Xinxiang 453007, People’s Republic of China; ‡ Department of Chemistry, 2536 Austin Peay State University, Clarksville, Tennessee 37044, United States; § Department of Physics and Astronomy, 5718 Vanderbilt University, Nashville, Tennessee 37235, United States

## Abstract

We employ real-space, real-time time-dependent density functional theory combined with Ehrenfest dynamics to investigate ultrafast intermolecular relaxation following inner-valence ionization in hydrated pyrrole. By treating nuclear and electronic dynamics on an equal footing, this approach captures their strong coupling and enables a fully time-resolved description of electronic excitation, charge transfer, ionization, and structural rearrangement. When an initial vacancy is created in the water 2 s^–1^ state, the system predominantly undergoes intermolecular Coulombic decay (ICD) and electron-transfer–mediated decay (ETMD), accompanied by significant intermolecular charge transfer between pyrrole and water. In contrast, fixed-nuclei simulations predict that ICD is the only active decay channel. For ionization of the pyrrole N 2s orbital, both ICD and Auger decay channels are observed, whereas neglecting nuclear motion leads to a qualitative change in the relaxation pathway, with Auger decay dominating exclusively. These results demonstrate that nuclear motion plays a decisive role in ultrafast relaxation dynamics. By dynamically modulating intermolecular distances and electronic couplings, it actively drives and reshapes the competition between decay channels, affecting both their efficiency and branching ratios. Overall, the relaxation pathways are governed by the interplay between the initial vacancy localization and coupled electron–nuclear dynamics, providing microscopic insight into energy- and charge-transfer processes in hydrated molecular systems.

## Graphical abstract

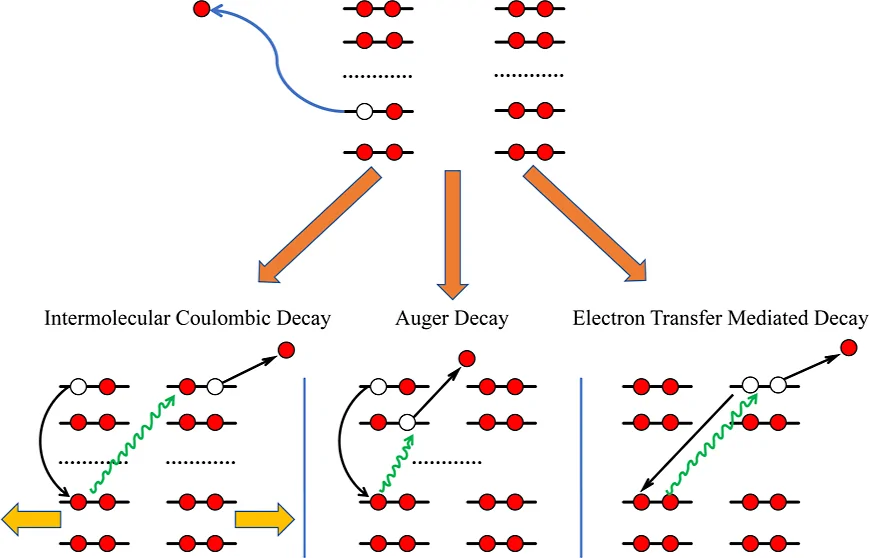

## Introduction

Inner-valence ionization is a fundamental process in radiation chemistry and photophysics, particularly in aqueous environments relevant to biological systems.^1–3^ It removes electrons from orbitals located between the core and outer-valence levels, producing excited cationic states that relax through ultrafast channels such as Auger decay, intermolecular Coulombic decay (ICD), and electron-transfer–mediated decay (ETMD).^4–7^ In hydrated clusters, solvent interactions decisively influence these pathways by modulating charge redistribution and energy transfer dynamics.^1,8^

Pyrrole, often regarded as a prototypical heterocycle for nucleobases, constitutes a key structural motif in many biologically important molecules, including vitamin B_12_, bile pigments such as bilirubin, the porphyrin ring of heme, and chlorophyll.^9,10^ Interactions between pyrrole and water provide a realistic model for pyrrole-based biomolecular environments. Recent experiments^11^ have revealed a double-ionization–induced decay process in hydrated pyrrole, accompanied by proton transfer that leads to the formation of deprotonated C_4_H_4_N^+^ and H_3_O^+^ cations.

On the theoretical side, Skitnevskaya et al.^1^ investigated the relaxation dynamics of inner-valence vacancies in hydrated pyrrole by computing single- and double-ionization spectra using the third-order algebraic diagrammatic construction [ADC(3)] and second-order ADC [ADC(2)] methods, respectively. Their results showed that, following inner-valence ionization of the water molecule, only nonlocal relaxation channels—ICD and ETMD—are energetically accessible. In contrast, for inner-valence ionization of the heterocycle, both local Auger decay and nonlocal ICD processes can occur. Subsequently, Kumar et al.^8^ examined the energetics of electron decay in inner-valence–ionized pyrrole–water pairs using complete active space second-order perturbation theory (CASPT2). Based on their calculated ionization potentials (IP) and double-ionization potentials (DIP), ionization of the N-2s inner-valence orbital of pyrrole can lead to three competing pathways: Auger decay, ICD, and proton transfer. For an O-2s vacancy, ICD and ETMD channels may be accessible alongside Auger decay. However, both theoretical studies were performed within the fixed-nuclei approximation. Previous investigations of related ICD processes^12,13^ have demonstrated that nuclear motion can play a crucial role in modulating decay efficiencies and influencing final-state distributions. Therefore, a comprehensive understanding of the ultrafast decay dynamics in hydrated pyrrole requires an explicit treatment of coupled electronic and nuclear dynamics, which is the central aim of the present work.

Recent theoretical studies have further emphasized that the competition between ultrafast electronic relaxation and proton motion is a general feature of inner-valence-ionized hydrogen-bonded systems. Real-time time-dependent density functional theory (TDDFT) simulations of the water dimer together with recent electronic-structure studies have demonstrated that nuclear motion can substantially modify decay branching ratios, relaxation time scales, and the interplay between ICD, ETMD, and proton-transfer processes, providing important motivation for extending these investigations to hydrated pyrrole.^14–16^

Molecular dynamics simulations based on the Born–Oppenheimer (BO) approximation are widely used to incorporate nuclear motion. However, such approaches are inherently limited to describing the postionization Coulomb explosion of fragment ions and cannot capture the decay processes initiated from the initial hole state. Consequently, they fail to address key ultrafast phenomena, including charge transfer, energy transfer, and the emission of decay electrons. In principle, the multiconfiguration time-dependent Hartree (MCTDH) method^17^ can provide a detailed description of these coupled electron–nuclear dynamics. Nevertheless, its computational cost scales exponentially with the number of degrees of freedom, severely restricting its applicability to systems with only a few active modes. Furthermore, its accuracy critically depends on the quality of the underlying potential energy surfaces, which makes it difficult to extend to complex systems such as hydrated pyrrole. As a result, existing theoretical approaches face significant challenges in describing the decay dynamics of hole states in cluster systems. Recently, we^18^ employed a TDDFT approach to investigate the decay dynamics of the O 2s ionized hole state in the water dimer. Our simulations revealed the coexistence of ICD and proton-transfer processes, demonstrating that real-time TDDFT provides a practical and efficient framework for exploring ultrafast decay dynamics in complex systems.

In the present study we employ a real-space, real-time TDDFT framework combined with Ehrenfest dynamics,^18^ in which electronic and nuclear degrees of freedom are treated on an equal footing.^19–21^ This approach enables a direct time-resolved characterization of ultrafast relaxation dynamics through simultaneous tracking of ionized-electron emission and the evolution of monomer charge distributions. Importantly, charge-transfer processes and nonadiabatic effects are naturally incorporated, while nuclear motion—particularly proton dynamics—can act as an effective gating mechanism for the decay pathways.^22^ Moreover, the framework is sufficiently general to investigate multiple competing ultrafast relaxation channels within a unified description, including Auger decay, ICD, and ETMD.

Although the present RT-TDDFT–Ehrenfest approach does not explicitly account for electronic decoherence or nuclear wavepacket branching, it provides a practical framework for describing the coupled electron–nuclear dynamics beyond the fixed-nuclei approximation. Its qualitative reliability for hydrogen-bonded systems is supported by previous RT-TDDFT–Ehrenfest studies of inner-valence ionization in the water dimer, where calculated ICD time scales were found to be in good agreement with experiment.^15^ Therefore, the present work is intended to reveal qualitative trends in the competition among ICD, ETMD, and Auger relaxation pathways rather than quantitative branching ratios for individual decay events.

Using this approach, we investigate the ultrafast relaxation dynamics of inner-valence–ionized hydrated pyrrole under different initial hole configurations, with particular emphasis on the role of nuclear motion in shaping the competing decay pathways. Our simulations provide microscopic insight into the interplay among Auger decay, ICD, and ETMD processes, and reveal how coupled electron–nuclear dynamics govern energy redistribution and charge migration in hydrated molecular clusters.

## Methods

The molecular dynamics in each set of simulations are modeled by real-time (RT) TDDFT on a real-space grid. The Kohn–Sham Hamiltonian has the following form

1 $${Ĥ}_{KS}\left(t\right)=-\frac{\hbar^{2}}{2m}\nabla^{2}+V_{ion}\left(r,t\right)+V_{H}\left[\rho \right]\left(r,t\right)+V_{XC}\left[\rho \right]\left(r,t\right)$$

Here, ρ is the electron density which is defined as the density sum over all occupied orbitals

2 $$\rho \left(r,t\right)=\sum_{k=1}^{N}2|\psi_{k}\left(r,t\right){|}^{2}$$

The coefficient 2 accounts for the presence of two electrons in each orbital due to electron spin degeneracy, while *k* denotes the quantum number for each orbital. The *V*_ion_ in eq 1 represents the external potential induced by ions, expressed using the norm-conserving pseudopotential centered on each ion as provided by Troullier and Martins.^23^ *V*_H_ denotes the Hartree potential, defined as

3 $$V_{H}\left(r,t\right)=\int \frac{\rho \left(r^{\prime },t\right)}{\left|r-r^{\prime }\right|}\;dr^{\prime }$$

representing the average electrostatic interaction resulting from electron repulsion. The last term, *V*_XC_, represents the exchange–correlation potential, which is approximated using the generalized gradient approximation (GGA) developed by Perdew et al.^24^ Prior to the time-dependent simulations, ground-state DFT calculations are performed to obtain the equilibrium electronic structure of the system, including the electron density, self-consistent Kohn–Sham orbitals, and total energy. With these quantities established as initial conditions, the time-dependent Kohn–Sham (TDKS) equations are then propagated in real time to evolve the Kohn–Sham orbitals ψ_*k*_(**r**, *t*) according to

4 $$i\hbar \frac{\partial \psi_{k}\left(r,t\right)}{\partial t}={Ĥ}_{KS}\left(t\right)\psi_{k}\left(r,t\right)$$

The equation is solved by time propagation

5 $$\psi_{k}\left(r,t+\delta t\right)\approx \exp\left[-\frac{i{Ĥ}_{KS}\left(t\right)\delta t}{\hbar }\right]\psi_{k}\left(r,t\right)$$

Eq 5 is approximated by a fourth-order Taylor expansion as follows

6 $$\psi_{k}\left(r,t+\delta t\right)\approx \sum_{n=0}^{4}\frac{1}{n!}{\left(\frac{-i\delta t}{\hbar }{Ĥ}_{KS}\left(t\right)\right)}^{n}\psi_{k}\left(r,t\right)$$

The time-propagation operator is applied iteratively for *N* time steps until the final simulation time *t*_final_ = *N*·δ*t* is obtained. A time step of δ*t* = 0.75 attosecond is employed in all simulations. In our RT-TDDFT calculations, the Kohn–Sham orbitals are represented on a uniform real-space grid. The accuracy of the simulations depends sensitively on the grid spacing, which serves as a key numerical parameter controlling spatial resolution. In the present work, we used a grid spacing of 0.22 Å, with 150, 140, and 140 grid points along the *x*, *y*, and *z* directions, respectively. The Kohn–Sham orbitals are set to zero at the boundaries of the simulation box. To suppress nonphysical reflections of the electronic density as ionized fragments approach the edges, a complex absorbing potential (CAP) is applied in the outer regions of the box. The CAP follows the form proposed by Manolopoulos^25^

7 $$-iw\left(x\right)=-\frac{i\hbar^{2}}{2m}{\left(\frac{2\pi }{\Delta x}\right)}^{2}f\left(y\right),,y=\frac{\left(x-x_{1}\right)}{\Delta x}$$

where *x*_1_ is the starting point of the absorption region, *x*_2_ is the end point, Δ*x* = *x*_2_ – *x*_1_, *c* = 2.62 is a numerical constant, *M* is the electron mass, and

8 $$f\left(y\right)=\frac{4}{c^{2}}\left(\frac{1}{{\left(1+y\right)}^{2}}+\frac{1}{{\left(1-y\right)}^{2}}-2\right)$$

When the molecule is ionized, the electron density propagates toward the boundaries of the simulation box, where it is absorbed by the CAP. The total number of electrons within the simulation box at time t is given by

9 $$N\left(t\right)=\int_{V}\rho \left(r,t\right)d^{3}x$$

where *V* denotes the volume of the simulation box. The total ionization of the system is then evaluated as *N*(0) – *N*(*t*). Nuclear motion is treated using the Ehrenfest method, in which nuclei evolve classically under the influence of time-dependent quantum forces. The force acting on the *i*-th nucleus is obtained as the gradient of the total energy with respect to the nuclear coordinate, expressed as

10 $$M_{i}\frac{d^{2}R_{i}}{dt^{2}}=\sum_{j\neq i}^{N_{ions}}\frac{Z_{i}Z_{j}\left(R_{i}-R_{j}\right)}{|R_{i}-R_{j}{|}^{3}}-\nabla_{R_{i}}\int V_{ion}\left(r,R_{i}\right)\rho \left(r,t\right)\;dr$$

where *M*_*i*_, *Z*_*i*_, and **R**_*i*_ are the mass, pseudocharge (valence), and position of the *i*th ion, respectively, and *N*_ions_ is the total number of ions. The differential equation propagates in time at each time step δ*t* in combination with the Verlet algorithm.

Figure 1 illustrates the optimized geometry of the hydrated pyrrole dimer, obtained using the B3LYP/aug-cc-pVTZ method. The distance between the oxygen and nitrogen atoms is 3.04 Å. In this configuration, the water molecule acts as an electron-density donor, while pyrrole serves as the electron-density acceptor. As discussed below, we investigate the electron-relaxation dynamics following inner-valence ionization of the hydrated pyrrole dimer. To represent the initial ionized state, a ground-state DFT calculation is first performed to obtain the electronic structure of the neutral system. An initial vacancy is then introduced by removing an electron from a specified molecular orbital, after which the system is propagated in real time using TDDFT. In the present calculations, a CAP is employed to effectively remove ionized electrons from the simulation domain. The charge of each monomer is evaluated by integrating the time-dependent electron density over regions centered on each molecular subunit.

**Figure 1:**
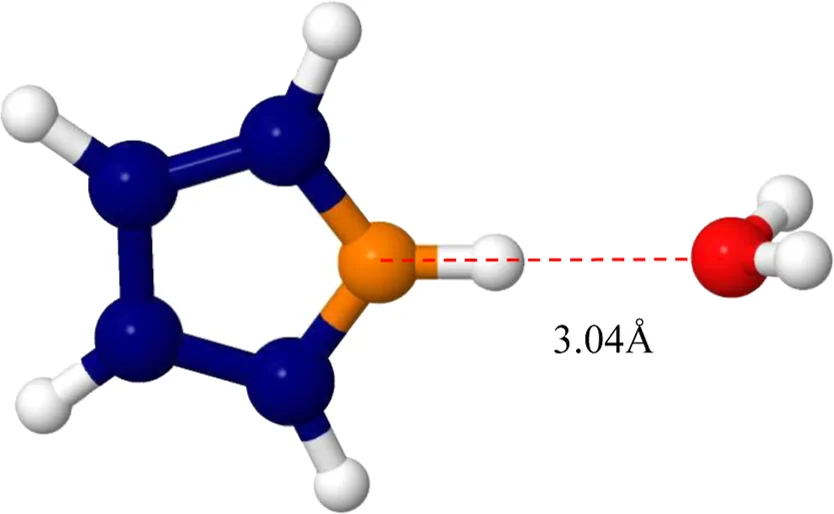
Geometric structure of the hydrated pyrrole dimer used in this study. The nuclear distance between O and N atoms is in angstroms.

## Results and Discussion

In this study, we investigate the possible decay channels that arise following inner-valence ionization of either the water molecule or the heteroatom in the pyrrole–H_2_O complex. Multiple dynamical trajectory simulations were performed, with the initial atomic velocities randomly sampled from a Boltzmann distribution at 300 K, a standard procedure for initializing nuclear motion.^18,19,26^ A total of 100 trajectories were propagated, with the number constrained by computational cost. When the initial hole for O 2 s^–1^ state was created on the water molecule, 50 trajectories were simulated, of which 40 led to ICD and 10 to ETMD. In contrast, when the hole for N 2 s^–1^ state was created on the pyrrole molecule, also 50 trajectories were performed, yielding 18 ICD and 32 Auger decay events. To elucidate the underlying relaxation mechanisms, ten representative trajectories are analyzed in detail below for each decay channel.

The pathway classifications discussed below correspond to the dominant relaxation behavior observed in each trajectory. Individual RT-TDDFT trajectories may contain weaker contributions from multiple relaxation channels because the coupled electron–nuclear dynamics continuously modifies the electronic structure. Consequently, the classification should be interpreted as identifying the dominant relaxation characteristics within the trajectory ensemble rather than mutually exclusive decay mechanisms.

### The Initial O 2 s^–1^ Vacancy

Figure 2 presents the time-dependent charge loss of the hydrated pyrrole dimer when the initial vacancy is created on the water molecule. Ten trajectories were randomly selected for each relaxation pathway, and the total charge loss, along with the charge loss on the water and pyrrole monomers, was calculated. The results for the ICD and ETMD processes are shown in Figure 2a,b, respectively. The average charge loss over the ten trajectories is also included in the figure for clarity. As shown in Figure 2a, at the initial time, the water molecule is cationic while the pyrrole molecule remains neutral. At 40 fs, the average charge loss on the water molecule reaches 1.0 and then remains nearly constant up to 200 fs, indicating that the water molecule approaches a monovalent cation state. In contrast, the average charge loss on the pyrrole molecule increases rapidly to 0.7 within the first 30 fs, followed by a slower increase to approximately 1.2 at 200 fs. These results indicate that most of the net charge loss occurs on the pyrrole molecule, whereas the water molecule retains its ionic character throughout the simulation. This behavior is consistent with the characteristic features of the ICD mechanism, confirming that the present simulation successfully captures the ICD process in the hydrated pyrrole dimer. The average maximum total charge loss is approximately 2.2, which is slightly higher than the theoretical expectation of 2. This deviation is reasonable, given the intrinsic limitations of TDDFT and the spatially defined CAP. The gradual increase in total charge loss arises from the finite time required for the ionized wave function to reach the CAP region, as discussed in our previous work.^18^ Notably, a pronounced increase in total charge loss is observed between 10 and 40 fs, suggesting that Coulombic decay predominantly occurs within this time interval.

**Figure 2:**
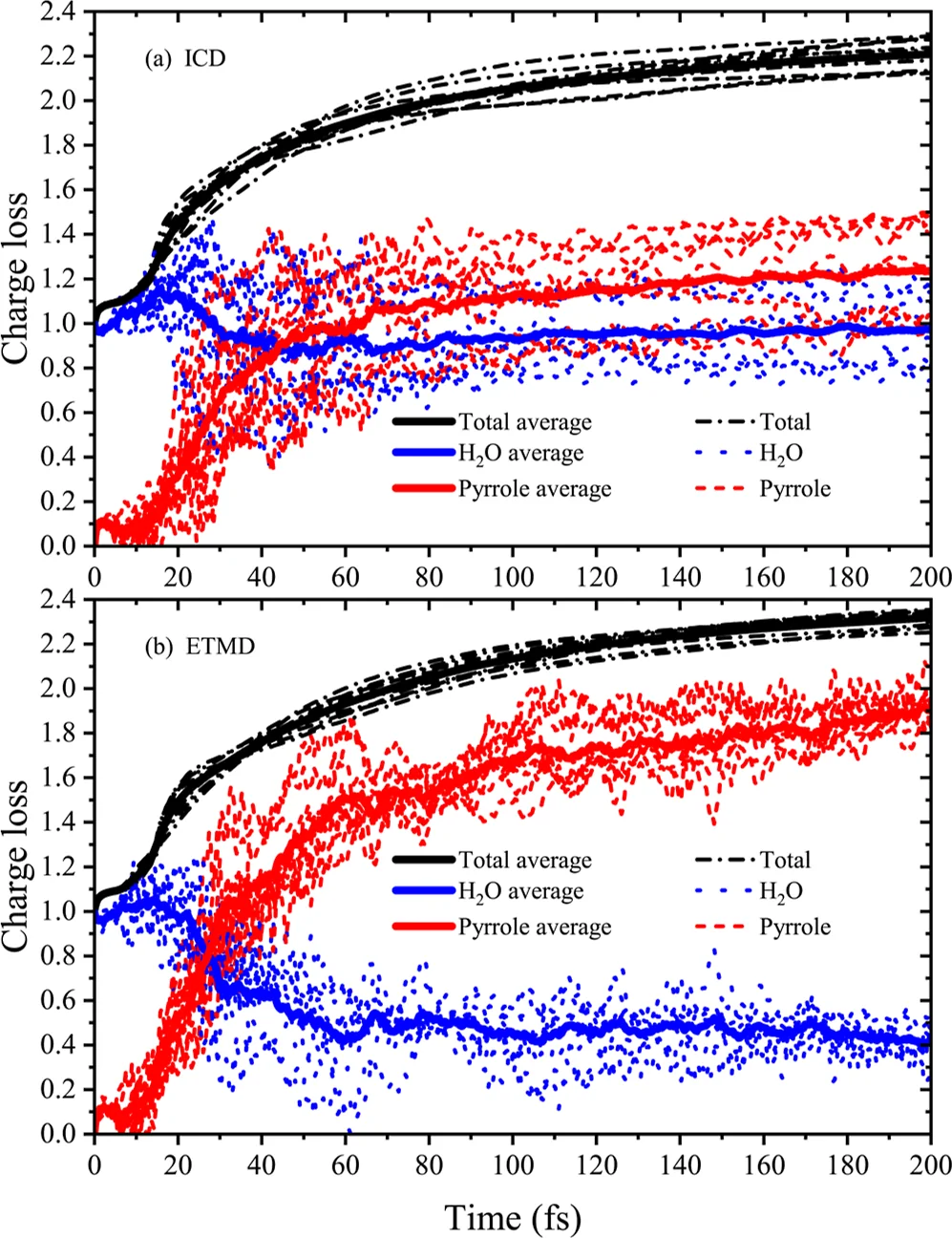
Time-dependent charge loss of pyrrole-water with the initial O 2 s^–1^ vacancy in water. (a) For the ICD process and (b) for the ETMD process.

The ionization of the oxygen 2s electron in water not only triggers the ICD process discussed above but also opens an alternative relaxation pathway—ETMD. Figure 2b shows the time-dependent charge loss of the hydrated pyrrole dimer for ten trajectories during this process. The average charge loss across these ten trajectories is also displayed in the figure. Between 0 and 200 fs, the average charge loss on the water molecule gradually decreases from 1.0 to 0.4. In contrast, the average charge loss on the pyrrole molecule increases from zero at the initial moment to 1.9 at 200 fs. By the end of the simulation, the water molecule becomes nearly neutral, while the pyrrole molecule has lost approximately two electrons, which is consistent with the ETMD mechanism. Although previous studies^1^ have suggested that ETMD typically occurs on a slower time scale than ICD, the use of a CAP in our simulations—employed to collect emitted electrons—precludes a precise determination of the exact onset and duration of the ETMD process. Consequently, only qualitative conclusions regarding its temporal evolution can be drawn in the present work.

As shown in Figure 2, the charge loss of the system reaches 2.3, exceeding the theoretically expected value of 2. To examine whether the CAP absorbs bound electrons, we apply a laser pulse that is not strong enough to induce ionization and monitor the electronic propagation of the system during this process. The collected charge by the CAP was found to be 0.002, indicating that the delocalization of the excited-state electron density has a negligible effect on the present evaluation of the charge loss. On the other hand, in the present calculations, the inner boundaries of the CAP region were set to ±12 Å, ±11 Å, and ±11 Å along the three spatial directions, respectively. We further tested the electron density loss using smaller CAP boundaries of ±11 Å, ±9 Å, and ±10 Å. By comparing the two sets of results, we found that with the larger CAP boundaries used in the present work, the average charge loss decreases by 0.062 for the ICD process and by 0.045 for the ETMD process. These results indicate that, during the dynamical evolution, the excited-state electron density can reach the CAP absorbing region, leading to an overestimation of the charge loss and implying that a larger simulation box would be required. However, employing a larger simulation box would substantially increase the computational cost and also require a longer propagation time for the emitted electrons to reach the box boundaries. Therefore, we did not adopt a larger simulation box in the present work.

Skitnevskaya et al.^1^ investigated the relaxation dynamics of inner-valence vacancies in hydrated pyrrole by computing single- and double-ionization spectra using the ADC(3) and ADC(2) methods, respectively. Their results showed that, following inner-valence ionization of the water molecule, only nonlocal relaxation channels—ICD and ETMD—are energetically accessible. This finding is fully consistent with the conclusions of the present study. In hydrated biomolecular clusters, inner-valence ionization of water molecules can initiate ICD, as previously confirmed in hydrated tetrahydrofuran clusters,^27^ consistent with the present findings. In addition to ICD, our simulations predict a competing decay channel—ETMD—in which the final state corresponds to a doubly ionized organic molecule.

To assess the impact of nuclear motion on the decay dynamics, we carried out additional simulations of the electronic dynamics within the fixed-nuclei approximation. The initial electronic state is identical to that employed in the simulations including nuclear motion; the only distinction is that the nuclear coordinates are frozen during the time propagation. Figure 3 displays the time-dependent charge loss of the pyrrole–water complex with the initial O 2 s^–1^ vacancy. The propagation was extended to 200 fs to ensure convergence of the electronic decay. The charge loss of the water molecule remains essentially constant at a value of 1 over the entire time interval, indicating that no additional ionization occurs on the water site after the initial inner-shell vacancy is created. In contrast, the pyrrole molecule exhibits a pronounced increase in charge loss, rising from 0 at early times to 0.9 at 200 fs. This behavior demonstrates that the relaxation of the water inner-shell vacancy predominantly proceeds via energy transfer to the neighboring pyrrole, which subsequently emits one electron. The results thus provide clear evidence for ICD process in the fixed-nuclei limit. This decay scenario is different from the dynamics observed when nuclear motion is included, where ICD and ETMD pathways compete. The comparison highlights the crucial role of nuclear motion in enabling and modulating the decay mechanisms in this system.

**Figure 3:**
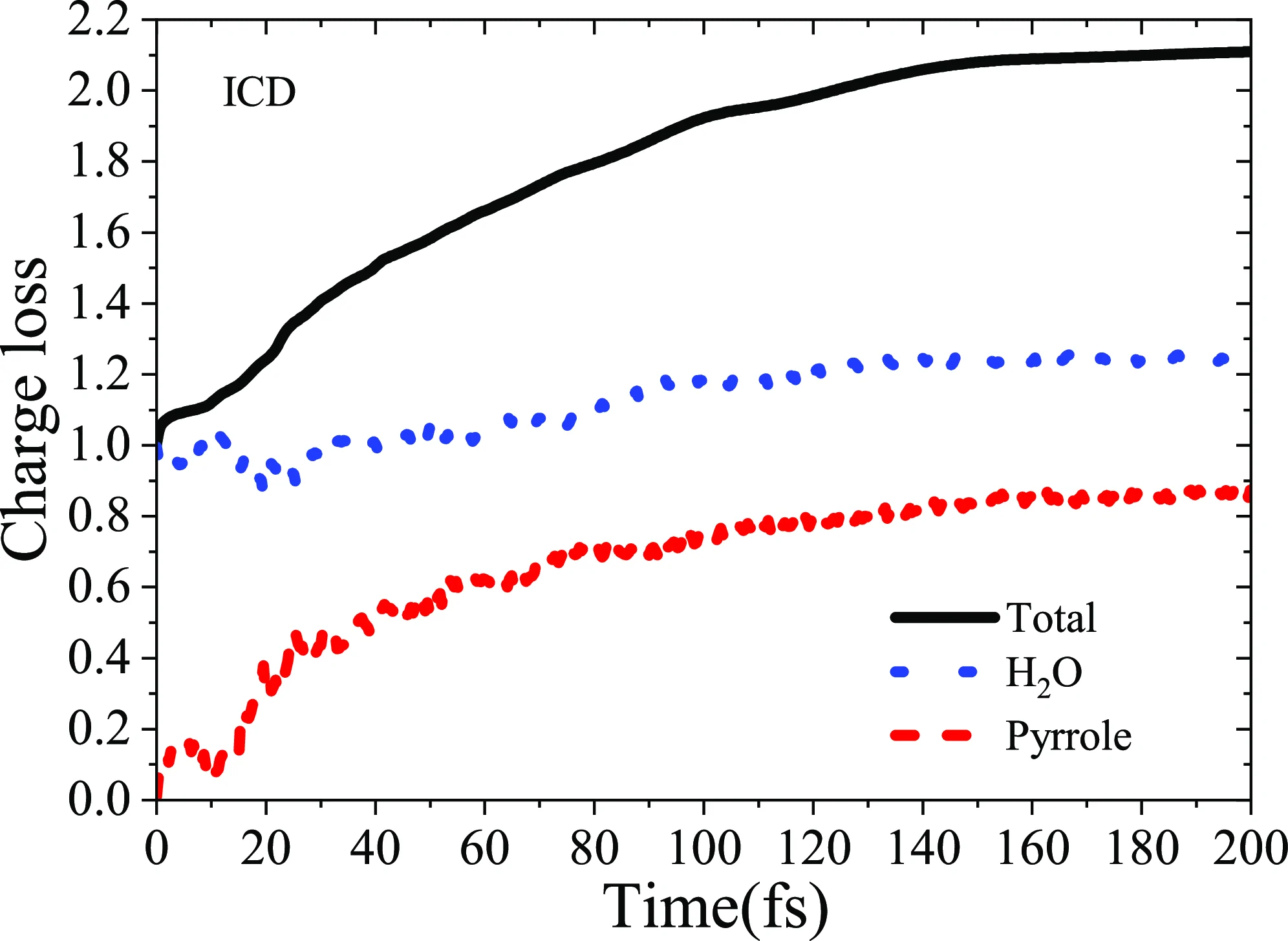
Time-dependent charge loss of pyrrole-water with the initial O 2 s^–1^ vacancy in water within the fixed-nuclei framework.

### The Initial N 2 s^–1^ Vacancy

We also examined the electron decay mechanism when the initial vacancy was located at the N 2s orbital of the pyrrole molecule. Similarly, we randomly selected ten trajectories from each set to analyze the charge loss on the water and pyrrole molecules. Figure 4a shows the time-dependent charge loss of pyrrole and water during the ICD process. For ease of discussion, the average charge loss is also presented in the figure. The average total charge loss gradually increased from 1.0 at 0 fs to 2.1 at 200 fs. From 0 to 55 fs, the average charge loss on the pyrrole molecule gradually increases to 1.3, after which it remains nearly constant for up to 200 fs. In contrast, the charge loss on water gradually increases from zero at the initial time to 0.8 at 200 fs. Since the majority of charge loss occurs on the water molecule, this behavior is characteristic of the ICD mechanism.

**Figure 4:**
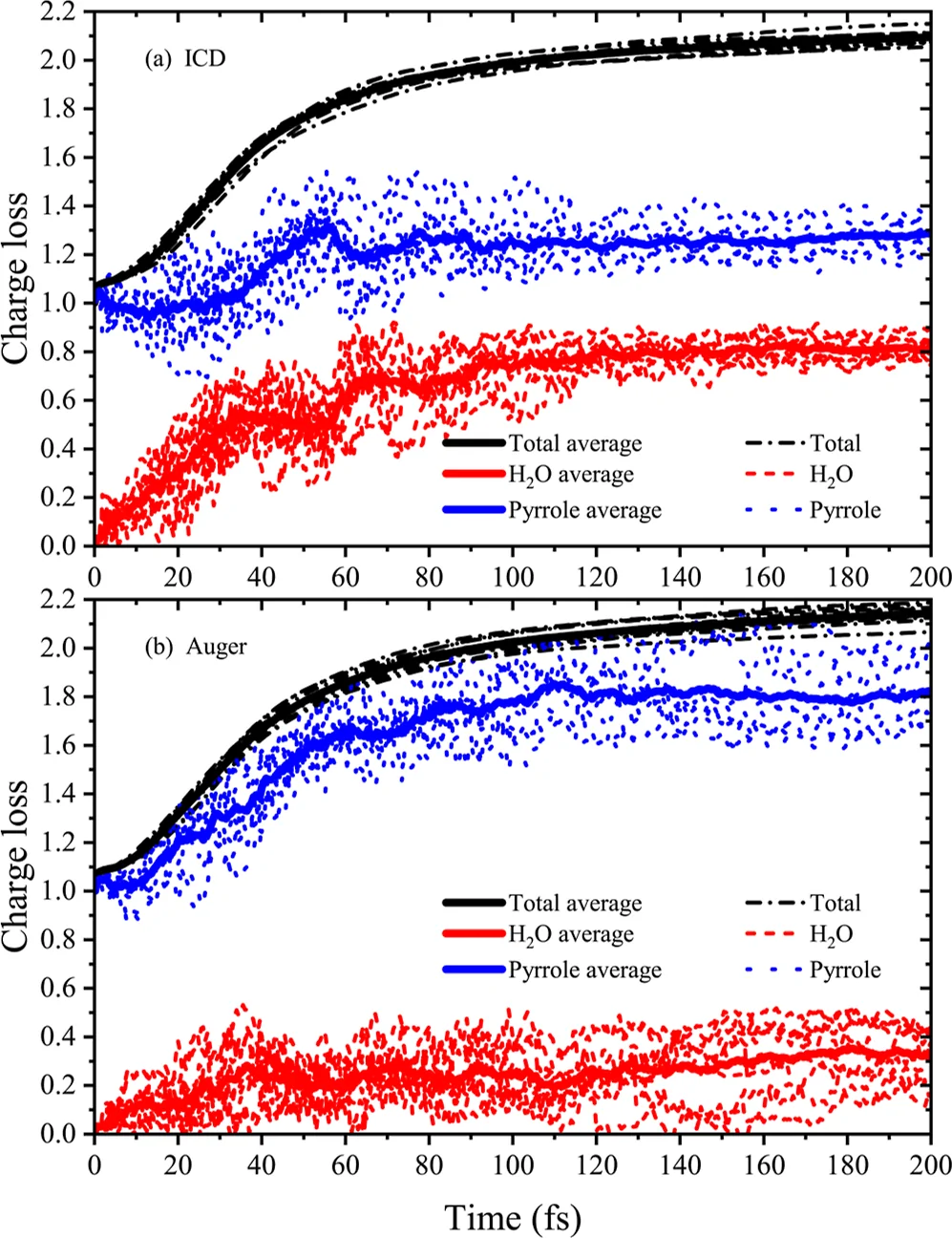
Time-dependent charge loss of pyrrole-water with the initial N 2 s^–1^ vacancy in pyrrole. (a) For the ICD process and (b) for the Auger process.

Figure 4b shows the time-dependent charge loss in pyrrole-water in which Auger decay occurs following inner-valence ionization of the N 2s in the pyrrole molecule. During the first 30 fs, the average total charge loss originates primarily from pyrrole, while the charge loss on water remained negligible. As the system evolves, a small but gradual increase in the charge loss on water is observed, reaching 0.3 within the simulated time window. After 30 fs, the average total charge loss continues to rise, eventually reaching a value of approximately 2.1 at 200 fs with pyrrole contributing about 1.8. This behavior indicates that the majority of electron emissions occur from pyrrole, consistent with the characteristics of Auger decay. The minor charge redistribution toward water likely reflects a weak intermolecular coupling during the relaxation. Therefore, these results confirm that under the current conditions, in the decay channels of inner-valence ionized pyrrole molecules caused by the N 2s electrons, Auger decay dominates.

Kumar et al.^8^ investigated the energetics of electron decay in N 2s ionized pyrrole–water pairs using the CASPT2. From their computed IP and DIP, they concluded that both ICD and Auger decay channels are energetically accessible. Our findings are consistent with the general conclusions of Kumar et al.^8^ and Skitnevskaya et al.^1^ However, Skitnevskaya et al. reported that Auger decay is the dominant relaxation pathway, regardless of whether the initial hole resides on O 2s or N 2s. In contrast, our real-time simulations show that when the vacancy originates from O 2s ionization, Auger decay is strongly suppressed and ICD becomes the primary relaxation channel. Only for N 2s ionization, Auger decay dominates, which is qualitatively consistent with the conclusion of Skitnevskaya et al. The differences between the present results and previous ADC/CASPT2 studies arise primarily from the treatment of the dynamics rather than from the underlying electronic-structure method. Whereas previous studies identified energetically accessible decay channels at fixed nuclear geometries, the present real-time simulations demonstrate that coupled electron–nuclear motion dynamically modifies intermolecular coupling and charge redistribution, thereby altering the relative importance of ICD, ETMD, and Auger decay and suppressing proton transfer on the simulated time scale.

Previous studies on hydrated clusters have demonstrated that proton transfer generally occurs when the initial hole is located on the proton-donating site of a hydrogen bond.^18,27^ Kumar et al.^8^ investigated the relaxation of a hole localized on the nitrogen atom in hydrated pyrrole. By extending the N–H bond and evaluating the IP of the neutral molecule using the CASPT2 method, they proposed that proton transfer may take place. In contrast, no proton transfer events were observed in our simulations across 50 trajectories, suggesting that the probability of such a process is relatively low. Nonetheless, the possibility of proton transfer cannot be completely excluded.

Figure 5 shows the time-dependent charge loss of pyrrole-water with the initial N 2 s^–1^ vacancy in pyrrole within the fixed-nuclei framework. As time increases from 0 to 200 fs, the charge loss on pyrrole rises from 1 to 1.9, whereas the charge loss on water remains in the range of 0–0.3 and overall stays nearly neutral. This behavior indicates that an Auger decay occurs in the system. Based on the above results that include nuclear motion, in addition to Auger decay, ICD also takes place following the decay of the N 2s vacancy. This confirms that nuclear motion drives the occurrence of the ICD process.

**Figure 5:**
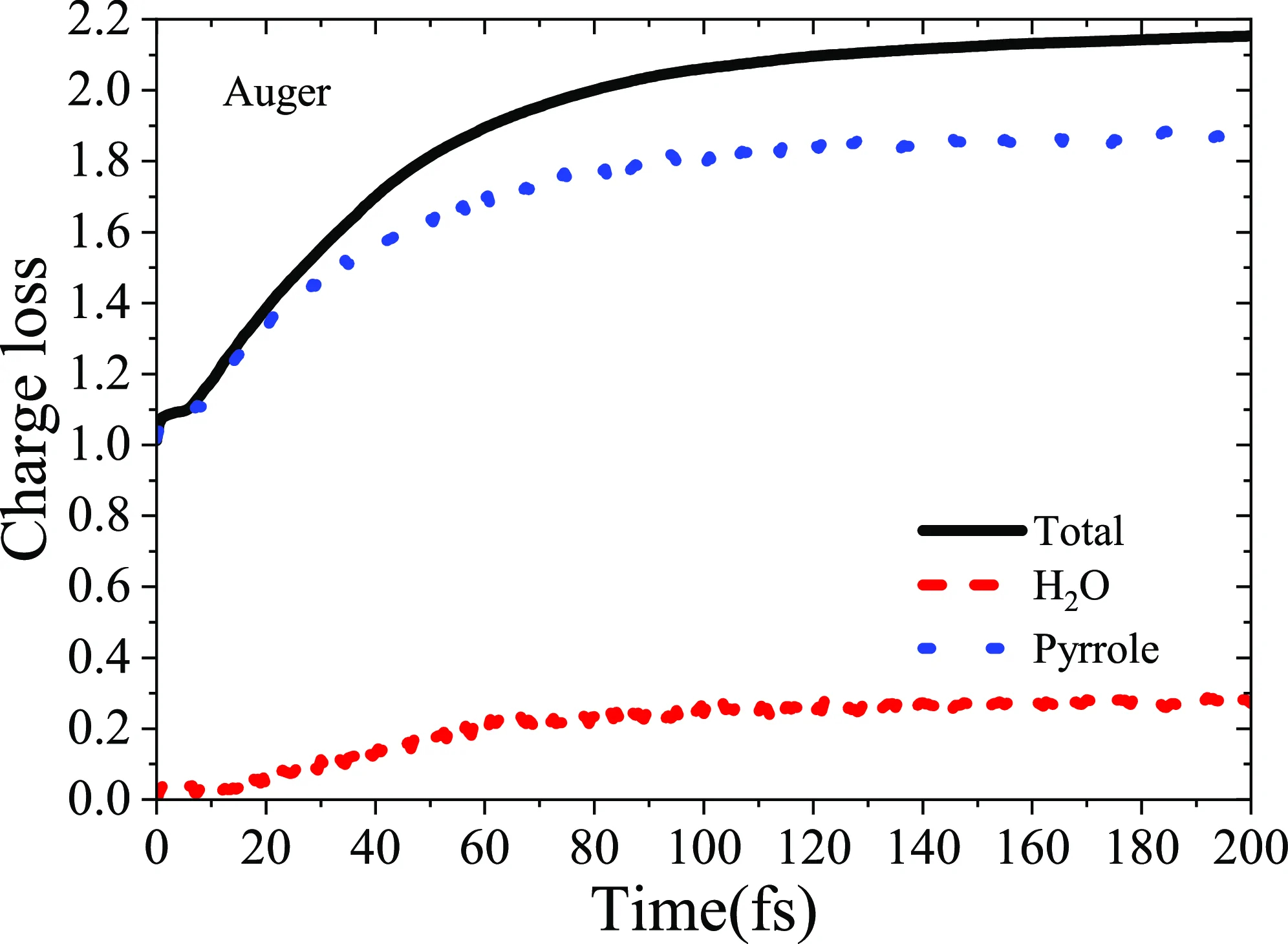
Time-dependent charge loss of pyrrole-water with the initial N 2 s^–1^ vacancy in pyrrole within the fixed-nuclei framework.

The observed dependence of the dominant relaxation pathway on the trajectory arises because the nuclear motion continuously modifies the hydrogen-bond geometry, intermolecular distance, and orbital overlap between water and pyrrole. These geometric changes alter the electronic coupling and energy alignment relevant to ICD, ETMD, and Auger decay, thereby changing their relative probabilities during the dynamics.

## Conclusions

We have investigated the ultrafast relaxation dynamics following inner-valence ionization in the hydrated pyrrole dimer by employing real-space RT-TDDFT combined with Ehrenfest dynamics. This approach enables a unified treatment of coupled electronic and nuclear motion, thereby capturing charge transfer, ionization, and structural evolution on the femtosecond time scale. The simulations, performed within the GGA for the exchange–correlation functional, reveal that the ensuing decay processes are driven by nuclear motion. Furthermore, the specific decay pathways are found to depend critically on the initial location of the valence vacancy.

When the initial vacancy is created on the water molecule, the system predominantly undergoes ICD, as observed in 40 out of 50 trajectories. In this process, energy is transferred to pyrrole, leading to secondary ionization and, ultimately, to Coulomb-driven dissociation. The remaining 10 trajectories proceed via ETMD, characterized by charge redistribution that drives pyrrole toward a dicationic state while partially neutralizing the water molecule. In contrast, when the initial vacancy is localized on the pyrrole moiety, the system predominantly undergoes Auger decay (32 out of 50 trajectories) with ICD events being relatively rare (18 out of 50 trajectories).

We additionally performed dynamic simulations within the fixed-nuclei approximation. In contrast to the simulations that explicitly include nuclear motion, the fixed-nuclei approach predicts qualitatively different decay pathways: the decay of an O 2s hole proceeds via ICD, whereas the decay of an N 2s hole is dominated by Auger decay. This comparison demonstrates that nuclear motion plays an essential role in shaping the decay dynamics and underscores the necessity of explicitly accounting for nuclear degrees of freedom in time-resolved simulations of inner-valence decay processes.

The pronounced sensitivity of these decay pathways to the initial vacancy location underscores their potential relevance to radiation-induced damage in aqueous and biological environments. The prevalence of ICD and ETMD implies an enhanced production of secondary low-energy electrons, which are known to play a crucial role in radiation chemistry and genotoxic processes. Overall, our results provide a microscopic framework for understanding intermolecular energy transfer dynamics in hydrated molecular systems, with implications for photostability and radiation therapy applications.

Our simulations utilized the mean-field Ehrenfest method, which computes nuclear dynamics on an averaged energy landscape weighted by electronic state populations. Other available approaches include trajectory surface hopping (TSH), where numerous independent classical paths model nuclear wave packet dynamics on separate Born–Oppenheimer potential surfaces, and the multiple spawning (MS) method, which expresses the nuclear wave function through Gaussian basis sets that evolve along classical trajectories. These three methods share a common feature: nuclear motion is described classically—although MS uses these classical paths as an auxiliary construct supporting quantum nuclear dynamics. As a result, all methods necessitate computing electronic characteristics (energies, energy gradients, coupling elements, etc.) at the instantaneous classical nuclear configurations throughout the simulation. ref 28 provides a detailed discussion of the advantages and drawbacks of these techniques. We plan to extend our computational framework to include TSH methodology and compare the relative effectiveness of Ehrenfest and TSH approaches.

It should be noted that the present conclusions are based on ensemble-level analysis of relaxation trends rather than on the prediction of exact quantum branching ratios for individual decay events. Following inner-valence ionization, RT-TDDFT describes the time-dependent electronic redistribution and charge-transfer processes, while Ehrenfest dynamics incorporates the feedback of nuclear motion beyond the fixed-nuclei approximation. Although electronic decoherence and nuclear wavepacket branching are not explicitly included, the present approach reveals how nuclear motion modifies intermolecular coupling and charge redistribution, thereby influencing the competition among ICD, ETMD, and Auger pathways. Therefore, Ehrenfest dynamics provides a practical framework for identifying qualitative trends in coupled electron–nuclear relaxation dynamics.

Another possibility is to maintain the Ehrenfest formalism while improving its precision. A core weakness of real-time TDDFT stems from the need to evaluate exchange–correlation functionals for strongly perturbed, nonequilibrium configurations far removed from ground-state conditions, despite these approximate functionals being calibrated using ground-state exchange–correlation data. This challenge can be overcome by recasting the Ehrenfest dynamics within a many-body basis set formulation that incorporates TDDFT-generated information—a strategy that has demonstrated improved accuracy in previous studies.^29^ We also plan to extend our studies to incorporate this approach.

## Data Availability

All data and code are available at https://github.com/kvvandy/tddft.
